# β-arrestin2 functions as a key regulator in the sympathetic-triggered immunodepression after stroke

**DOI:** 10.1186/s12974-018-1142-4

**Published:** 2018-04-10

**Authors:** Huan Wang, Qi-Wen Deng, Ai-Ni Peng, Fang-Lan Xing, Lei Zuo, Shuo Li, Zheng-Tian Gu, Fu-Ling Yan

**Affiliations:** Department of Neurology, Affiliated ZhongDa Hospital, School of Medicine, Southeast University, Dingjiaqiao 87, Nanjing, 210009 People’s Republic of China

**Keywords:** Stroke, Sympathetic pathway, Immunodepression, β-arrestin2, NF-κB

## Abstract

**Background:**

Stroke-induced immunodeficiency syndrome (SIDS) is regarded as a protective mechanism for secondary inflammatory injury as well as a contributor to infection complications. Although stroke-induced hyperactivation of the sympathetic system is proved to facilitate SIDS, the involved endogenous factors and pathways are largely elusive. In this study, we aim to investigate the function of beta-arrestin-2 (ARRB2) in the sympathetic-mediated SIDS.

**Methods:**

Splenic ARRB2 expression and the sympathetic system activity were detected after establishing transient models of middle cerebral artery occlusion (MCAO). In addition, a correlation between ARRB2 expression and the sympathetic system activity was analyzed using a linear correlation analysis. Any SIDS reflected in monocyte dysfunction was investigated by measuring inflammatory cytokine secretion and neurological deficit scores and infarct volume were tested to assess neurological outcome. Further, ARRB2 expression in the monocytes was knocked down in vitro by siRNAs. Following the stimulation of noradrenaline and lipopolysaccharide, cytokine secretion and the nuclear factor-κB (NF-κB) pathway were evaluated to gain insight into the mechanisms related to the contribution of ARRB2 to adrenergic-induced monocyte dysfunction.

**Results:**

Splenic ARRB2 expression was significantly increased after stroke and also showed a significant positive correlation with the sympathetic system activity. Stroke-induced monocyte dysfunction resulted in an increase of the interleukin-10 (IL-10) level as well as a decrease of the interleukin-6 (IL-6), tumor necrosis factor-α (TNF-α) and interleukin-1β (IL-1β) levels. Also, blockade of adrenergic-activity significantly reversed these cytokine levels, and blockade of adrenergic-activity improved stroke-induced neurological results. However, the improved neurological results had no significant correlation with ARRB2 expression. Furthermore, the in vitro results showed that the deficiency of ARRB2 dramatically repealed adrenergic-induced monocyte dysfunction and the inhibition of NF-κB signaling phosphorylation activity.

**Conclusions:**

ARRB2 is implicated in the sympathetic-triggered SIDS, in particular, monocyte dysfunction after stroke. Accordingly, ARRB2 may be a promising therapeutic target for the immunological management of stroke in a clinic.

## Background

Acute ischemic stroke is followed by profound immunoreactions, including an inflammatory response and subsequent immunodepression [1, 2]. In addition, an immunodepressive reaction is called stroke-induced immunodeficiency syndrome (SIDS) and is reflected in monocyte dysfunction and lymphocytopenia [3–5]. As SIDS contributes to brain repair mechanisms and infection complications, the underlying inflammatory mechanisms are under intensive investigation [6, 7].

Evidence is accumulating that the sympathetic system is excessively activated after stroke, which could facilitate SIDS [8, 9]. Catecholamines are mediators between the injured brain and immune cells [10, 11]. In addition, beta-adrenergic receptor blockers were shown in experimental studies to normalize stroke-induced immunological impairment [12, 13]. Yet, despite evidence that the sympathetic system plays a significant role in SIDS, the endogenous factors and pathways involved are largely elusive.

Beta-arrestin2 (ARRB2) is a ubiquitously expressed protein that was first described for its key role in desensitizing G-protein-coupled receptors (GPCRs). It is suggested that it regulates multiple intracellular signaling pathways [14–16]. Moreover, the role of ARRB2 in the modulation of inflammatory response has received increasing attention [17, 18]. In this study, it is hypothesized that ARRB2 can be involved in the affected pathways of sympathetic-triggered SIDS. Hopefully, clarifying the immunological function of ARRB2 in SIDS can contribute to the identification of novel therapeutic targets for the devastating condition of stroke.

Therefore, the current study firstly attempts to verify whether ARRB2 expression is increased after stroke, and further evaluates the correlation between ARRB2 expression and the sympathetic system activity through establishing models of middle cerebral artery occlusion (MCAO). Secondly, the effect of ARRB2 on intracellular signal transduction in β-adrenoreceptor mediated immunodepression is analyzed by the deficiency of ARRB2 in vitro.

## Methods

### Induction of experimental stroke model

Adult Sprague-Dawley male rats weighing 240–270 g (Qinglongshan Laboratory Animal Center, Nanjing, China) were used in all experiments. All animal experimental procedures and animal care were approved by the Ethics Committee of Southeast University, China and were conducted in accordance with the guidelines of the National Institutes of Health on the care and use of animals. Experimental brain ischemia was induced by transient filament occlusion of the dexter middle cerebral artery (MCA) for 60 min. In a sham group, the MCA was also exposed, but without occlusion. One group of rats with MCAO was intraperitoneally injected with 10 mg/kg propranolol (Sigma-Aldrich, St. Louis, MO, USA) dissolved in saline (immediately before MCAO, 4 and 8 h after MCAO) to inhibit the activation of the sympathetic nervous system [19]. An equivalent volume of saline was injected into another group of MCAO rats as well as the sham group. Experimental groups were randomly assigned as three groups: sham (sham operation) + saline (*n* = 8), MCAO + saline (*n* = 8), and MCAO + propranolol (*n* = 8).

### Neurological evaluation

Three days after reperfusion, the rats from each group were evaluated for neurological deficit. A neurological behavior assessment was blindly performed according to the Longa score methods [20]. The neurological function was graded on a scale of 0 to 4; 0, no neurologic deficit; 1, fail to extend forepaw on lifting the whole body by tail; 2, counterclockwise circling; 3, failure to the left or no autonomous motor activity; and 4, fail to walk spontaneously and response to external noxious stimulus.

### Assessment of infarct volume

After conducting a neurological evaluation, the brains were removed and sectioned at 2 mm intervals in the coronal plane. These slices were stained in a 2% solution of 2,3,5-triphenyltetrazolium chloride (TTC) for 30 min at 37 °C and then were fixed in a 4% solution of paraformaldehyde overnight. ImageJ software, version 13 (NIH, Bethesda, Maryland, USA) was used to analyze infarct volumes (corrected for edema) after TTC images of brain sections were digitized, as previously described [21, 22]. It should be mentioned that infarct percentage was calculated as [contralateral hemisphere volume − (ipsilateral hemisphere volume − infarct volume)] /contralateral hemisphere volume * 100%.

### Isolation of splenic macrophage

Spleens were removed and ground to pass them through a 50 μm nylon mesh (BD Falcon, Bedford, MA, USA). The prepared single-cell suspension was washed using RPMI1640 (Invitrogen Co., CA, USA) and was counted. Then, the cells were re-suspended (1 × 10^6^ cells/mL) for cell sorting. CD11b^+^ monocytes/macrophages were isolated using an EasySep Positive Selection Kit (STEMCELL Technologies Inc., Canada) according to the manufacturer’s instructions. The purified monocytes or macrophages were cultured in culture dishes with 50 ng/mL of lipopolysaccharide (LPS) for 6 h [23].

### Deficiency of ARRB2 by small interfering RNAs (SiRNAs) transfection in vitro

In order to achieve high transfection efficiency, THP-1 monocytes (The Cell Bank of Type Culture Collection of Chinese Academy of Sciences, Shanghai, China) were applied for deficiency of ARRB2. Cells were cultured in RPMI1640 (Invitrogen Co., CA, USA) supplemented with 10% heat-inactivated fetal bovine serum (FBS). SiRNAs targeting ARRB2 (LV3-β-arrestin2, 5′-GGACACCAACCTCATTGAATT-3′) and its empty vector (LV3-NC, 5′-TTCTCCGAACGTGTCACGT-3′) (GenePharma Co., Ltd., Shanghai, China) were added to cell suspension and incubated overnight. Transfection was undertaken with 5 μg/ml of polybrene (Invitrogen Co., Shanghai branch, China).

Stably transfected cells were established by 10 μg/mL of puromycin (Sigma-Aldrich, St. Louis, MO, USA) for 3 days. Cells were examined under a fluorescence microscope to determine transfection efficiency. Subsequently, they were differentiated into macrophages with 1.28 μM of phorbol myristate acetate (PMA) (5 × 10^5^ cells/well in 24-well plate, MultiSciences, China) for 48 h. After that, 50 ng/ml of LPS for 6 h was applied to stimulate cells to simulate bacteria invasion [23]. Meanwhile, 100 μM of noradrenaline (Sigma-Aldrich, St. Louis, MO, USA) was used to stimulate cells in order to simulate adrenergic activity [24].

### ELISA assays

Rat plasma and cell culture supernatants were collected and frozen at a temperature of 80 °C where necessary. Commercial ELISA kits (JoyeeBiotechnics Co., Ltd., Shanghai, China) were used for the quantitative analysis of the adrenergic neurotransmitter production (adrenaline = A and noradrenaline = NA) and the secretion of interleukin 6 (IL-6), tumor necrosis factor alpha (TNF-α), interleukin-1β (IL-1β), interleukin 8 (IL-8), and interleukin 10 (IL-10). Optical density was measured at 450 nm, and concentrations were calculated by referring to a standard curve.

### Protein isolation and western blot analytical technique

Protein was isolated in 300 μL of radio-immunoprecipitation assay (RIPA) lysis buffer with 3 μL of phenylmethane sulfonyl fluoride (PMSF) and protease inhibitor (Abcam Co., Bristol, UK), and was measured by the bicinchoninic acid (BCA) protein assay (Beyotime, Shanghai, China). The total protein (20 μg) was electrophoresed in SDS-PAGE with 8–10% polyacrylamide gels and then was transferred to polyvinylidene difluoride (PVDF) membranes (Merck Millipore, Billerica, MA, USA). Next, the membranes were blocked for 1 h with 5% (*w*/*v*) non-fat milk in Tris-buffered saline Tween-20 (TBST). The membranes were then incubated with rabbit primary antibodies overnight (anti-β-arrestin2, anti-NF-κB p65, anti-IκBα, anti-phosphor IκBα, anti-β-actin, CST, USA) at 4 °C. After washing and incubation with HRP-coupled goat anti-rabbit antibody (Abcam, Bristol, UK) for 2 h at room temperature, the membranes were visualized using chemiluminescence (Amersham, Uppsala, Sweden) and were analyzed using an automatic digital gel imaging analysis system (Peiqing JS-780, Shanghai Peiqing Science and Technology Co., Ltd., Shanghai, China).

### RNA extraction and RT-qPCR analysis

RNA was isolated with the TRIzol Reagent (Invitrogen Co., CA, USA), and the purity of RNA was checked using a NanoDrop spectrophotometer (Thermo Scientific, MA, USA). Reverse transcription was performed using HiScript 1st Strand cDNA Synthesis Kit (Vazyme Biotech Co., Ltd., Nanjing, China) with a polymerase chain reaction (PCR) (Eppendorf, Hamburg, Germany). RT-qPCR was carried out on the StepOnePlus™ Real-Time PCR system (Thermo Fisher Scientific, Cleveland, USA) using AceQ™ qPCR SYBR Green Master Mix (Vazyme Biotech Co., Ltd., Nanjing, China). Primers for RT-PCR were described in Table 1, which were purchased from Generay Biotech Company (Shanghai, China). The results were presented as the number of target gene copies per 35 copies. Analysis was performed using β-actin as a housekeeping gene standard.

**Table 1:** All gene primer sequences (Generay Biotech Co., Ltd., Shanghai, China) applied in the qPCR analysis

Gene (GenBank)		Primer sequence (5′-3′)
IL-6 (NM_000600.3)	Forward	CAGACAGCCACTCACCTC
	Reverse	CTCAAACTCCAAAAGACCAG
IL-10 (NM_000572.2)	Forward	GGAGAACCTGAAGACCCT
	Reverse	TGATGAAGATGTCAAACTCACT
IL-1β (NM_000576.2)	Forward	ACCACCACTACAGCAAGG
	Reverse	AAAGATGAAGGGAAAGAAGG
IL-8 (NM_000584.3)	Forward	GCATAAAGACATACTCCAAACC
	Reverse	AAACTTCTCCACAACCCTCT
TNF-α (NM_000594.3)	Forward	TGTAGCAAACCCTCAAGC
	Reverse	GGACCTGGGAGTAGATGAG
NF-κB (NM_001165412.1)	Forward	CCACAAGCAAGAAGCTGAAG
	Reverse	AGATACTATCTGTAAGTGAACC
IκBα (NM_020529.2)	Forward	ACACTAGAAAACTTCAGATGC
	Reverse	ACACAGTCATCATAGGGCAG
ARRB2 (NM_001257331.1)	Forward	TGTGGACACCAACCTCATTG
	Reverse	TCATAGTCGTCATCCTTCATC
β-actin (NM_001101.3)	Forward	GCACCACACCTTCTACAATGAG
	Reverse	ATAGCACAGCCTGGATAGCAAC

### Statistical analysis

Each experiment was undertaken in triplicate. All values were expressed as means ± standard deviations (SDs) and were statistically analyzed using SPSS software, version 17.0 (SPSS Inc., Chicago, IL, USA). A one-way analysis of variance (one-way ANOVA) and a Student’s *t* test were undertaken for comparisons between the groups. If the data was a non-continuous variable, the non-parametric Mann-Whitney *U* test was performed to investigate differences between the groups. The linear correlation analysis was applied to explore the relationship between ARRB2 and the adrenergic activity using GraphPad Prism6 (GraphPad Software Inc., San Diego, CA, USA). Furthermore, the partial correlation analysis was applied to evaluate the relationship between ARRB2 and neurological deficits or infarct volume using SPSS software to control the effect of the adrenergic activity. Significance was accepted for all analyses at *P* < 0.05.

## Results

### Hyperactivation of the sympathetic system and increasing ARRB2 expression after stroke

**Fig. 1:**
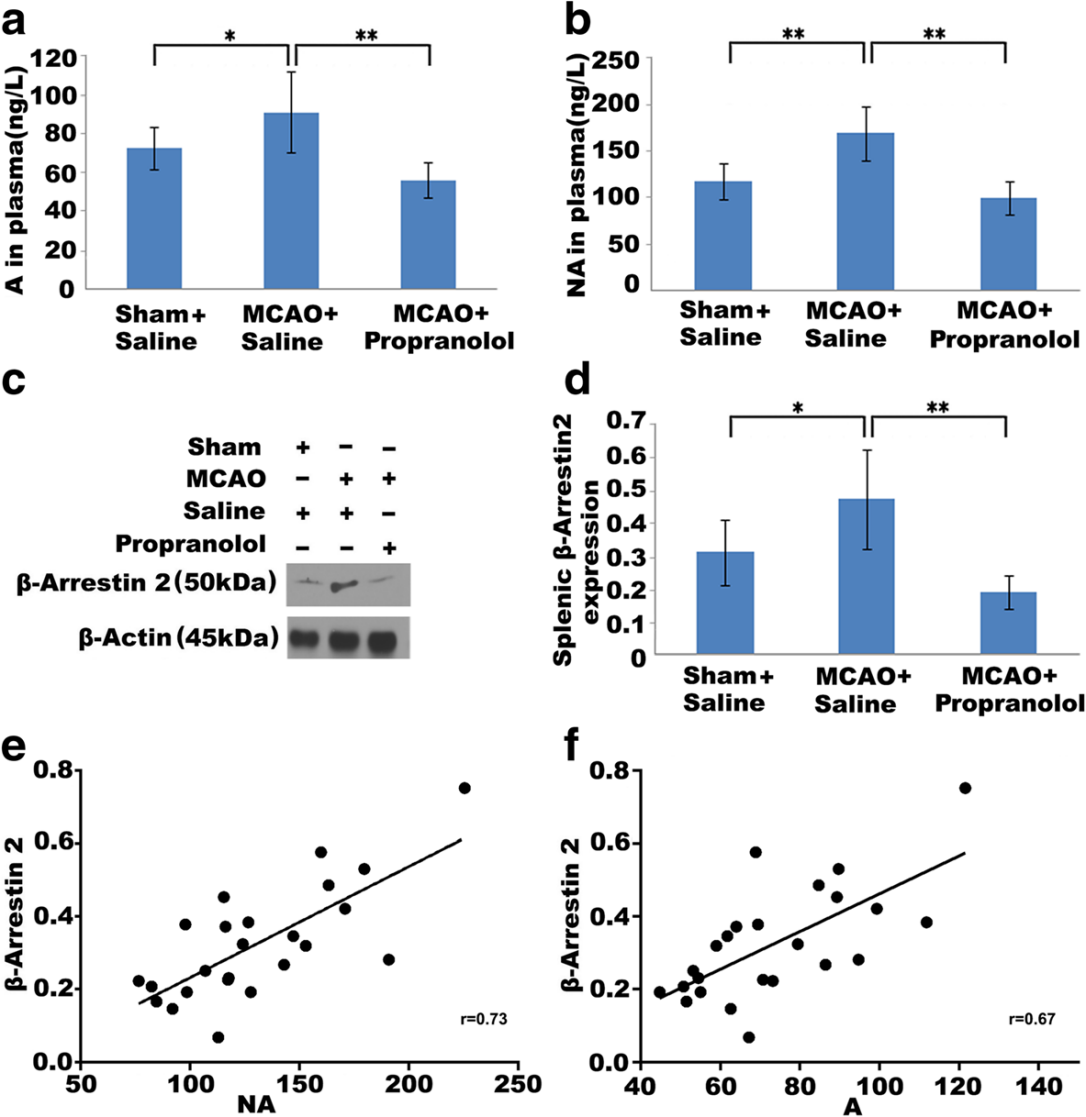
ARRB2 expression is increased in spleen after stroke and has a positive correlation with the sympathetic system activity. 3 days after MCAO, the plasma levels of A (**a**) and NA (**b**) in sham-operated and saline or propranolol (β-blocker)-treated MCAO models were detected by ELISA. In addition, A and NA levels of the MCAO + saline group were significantly higher than those of the sham + saline and the MCAO + propranolol groups. **c** Representative western blot image shows splenic ARRB2 expression of the three groups. **d** Densitometry analysis is shown as graph bars for ARRB2 expression level normalized with β-actin. Splenic ARRB2 expression in the MCAO + saline group was remarkably higher than in the sham + saline and the MCAO + propranolol groups. *n* = 8 animals per each group, and each experiment was independently replicated three times. *P < 0.05, and **P < 0.01 by one-way ANOVA. Analysis of linear correlation between splenic ARRB2 expression and the plasma level of NA (**e**), as well as A (**f**) indicates a strong correlation between splenic ARRB2 expression and the sympathetic system activity. The values of the obtained correlation coefficient are *r* = 0.73 (*P* < 0.01) and *r* = 0.67 (*P* < 0.01), respectively. A = adrenaline, NA = noradrenaline

Three days after stroke, the plasma level of A of the MCAO + saline group was significantly higher than that of the sham + saline group (*P* < 0.05). Especially, the NA level was substantially increased in the MCAO + saline group compared with the sham + saline group (*P* < 0.01). However, both A and NA levels could be decreased by propranolol treatment in MCAO compared with MCAO + saline (*P* < 0.01, *P* < 0.01) (see Fig. 1a,b). Consequently, these results demonstrated that the sympathetic system was remarkably hyperactivated after stroke, and propranolol could inhibit the sympathetic system hyperactivation.

On the other hand, ARRB2 expression of the MCAO + saline group was markedly higher than that of the sham + saline group (*P* < 0.05). However, stimulation by propranolol distinctly suppressed ARRB2 expression in spite of MCAO establishment (*P* < 0.01), as shown in Fig. 1c,d. The linear correlation between ARRB2 expression and sympathetic neurotransmitter release (by treating A and NA levels separately) showed that significant positive correlations were found between ARRB2 expression and both A (*r* = 0.67, *P* < 0.01) and NA levels (*r* = 0.73, *P* < 0.01) (see Fig. 1e,f).

### Inhibition of the sympathetic system attenuates stroke-induced splenic monocyte dysfunction

**Fig. 2:**
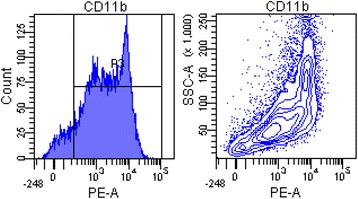
Splenic monocytes/macrophages isolated from spleen at 3 days after MCAO. Representative FACS results of cell purity analysis. Cells were isolated using the EasySep Positive Selection Kit

**Fig. 3:**
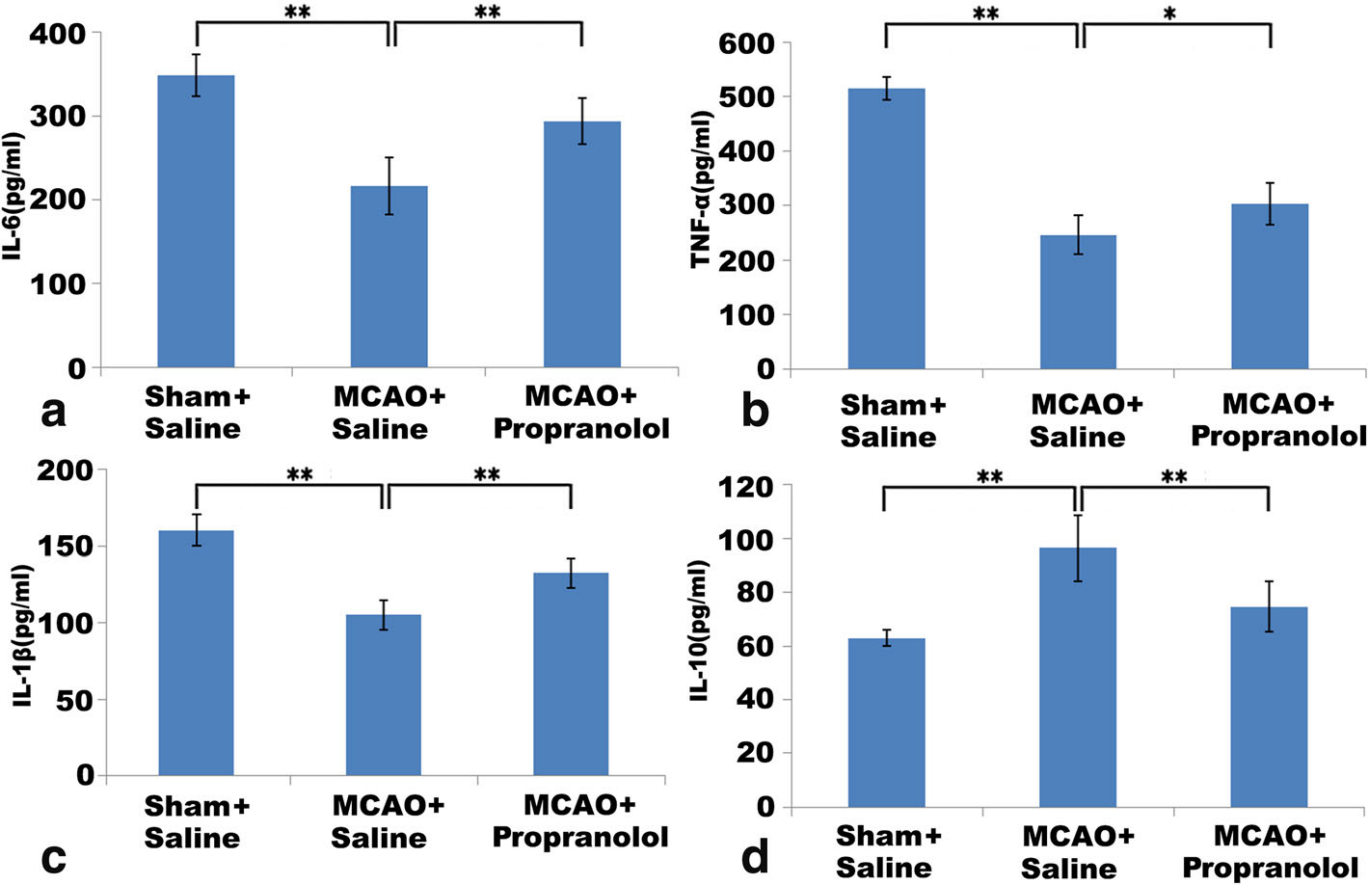
Effects of the sympathetic system activity on the levels of cytokine secreted by splenic monocytes/macrophages 3 days after MCAO. The secretion of cytokines was detected by ELISA. The stimulation of LPS induced high secretion of pro-inflammatory cytokines including IL-6 (**a**), TNF-α (**b**), IL-1β (**c**), and anti-inflammatory cytokine IL-10 (**d**) from splenic monocytes in the sham + saline group. The secretion of these pro-inflammatory cytokines (**a**–**c**) in the MCAO + saline group was significantly reduced compared with the sham + saline group. In addition, IL-10 level was significantly increased in the MCAO + saline group (**d**). However, blockade of the sympathetic activity substantially raised secretion levels of pro-inflammatory cytokine (**a**–**c**) and decreased IL-10 (**d**) level despite MCAO establishment. *n* = 8 animals per group, and each experiment was independently replicated three times. *P < 0.05 and **P < 0.01 by one-way ANOVA

As illustrated in Fig. 2, the purity of splenic monocytes/macrophages isolated from spleens of each group was over 90%. With stimulation of LPS, the expression levels of pro-inflammatory cytokines including IL-6, TNF-α, and IL-1β in the MCAO + saline group were remarkably reduced compared with the sham + saline group (*P* < 0.01, *P* < 0.01, *P* < 0.01, as shown in Fig. 3a–c). In addition, the expression level of IL-10 was greatly increased in the MCAO + saline group compared with the sham + saline group (*P* < 0.01, see Fig. 3d). However, as displayed in Fig. 3a–c, blockade of the sympathetic activity significantly increased these pro-inflammatory cytokine levels in spite of MCAO establishment (*P* < 0.01, *P* < 0.05, *P* < 0.01). Moreover, IL-10 expression was substantially decreased by blockade of the sympathetic activity in spite of MCAO establishment (*P* < 0.01, see Fig. 3d). These results demonstrate that MCAO establishment induces dysfunction of splenic monocytes, and blockade of the sympathetic activity remarkably reversed the alteration.

### Inhibition of sympathetic activity reduces infarct volume in acute experimental stroke

**Fig. 4:**
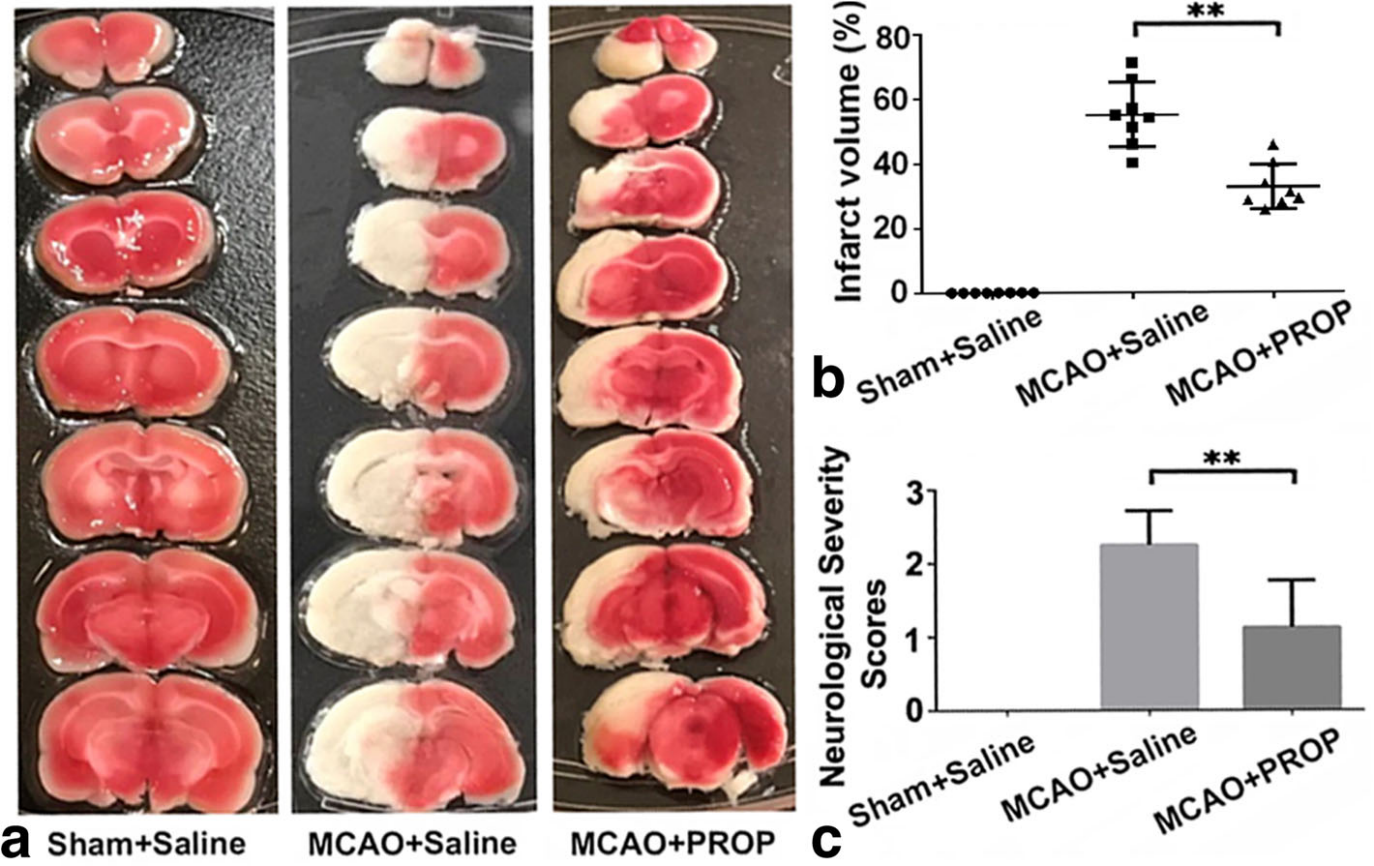
MCAO models were successfully established and effects of blockade of the sympathetic system on infarct volume and neurological deficits. **a** Representative coronal sections of brain infarct stained with TTC at 3 days after MCAO. White staining indicates infarction, and red staining indicates normal tissue. **b** Quantification of infarct volume. Infarct volumes were smaller in the MCAO + PROP group than in the MCAO + saline group. **c** Neurological deficit scores at 3 days after MCAO. In the MCAO + PROP group, neurological scores were considerably decreased compared with the MCAO + saline group. *n* = 8 animals per group, and each experiment was independently replicated three times. *P < 0.05 and **P < 0.01 by one-way ANOVA. PROP = propranolol

We further determined whether the inhibition of sympathetic activity can protect the brain against ischemic injury. As depicted in Fig. 4a, infarct volumes were prominently measured in the MCAO groups and the sham + saline group. Infarct volume in the propranolol-treated MCAO group was 32.59% ± 6.89%, which was notably reduced compared with the saline-treated MCAO group (54.99% ± 10.03%, *P* < 0.01), as shown in Fig. 4a,b. With the controlling of the effect of the sympathetic activity, there was no significant correlation between the infarct volume and ARRB2 expression (*P* = 0.364).

Additionally, we evaluated the neurological deficit in each group (see Fig. 4c). The neurological deficit was significantly observed in the MCAO-operated rats. However, propranolol treatment dramatically reduced the score in spite of MCAO establishment (*P* < 0.01). It is noteworthy that no significant correlation was found between the neurological deficit and ARRB2 expression (*P* = 0.356).

### Downregulation of ARRB2 expression by siRNA

**Fig. 5:**
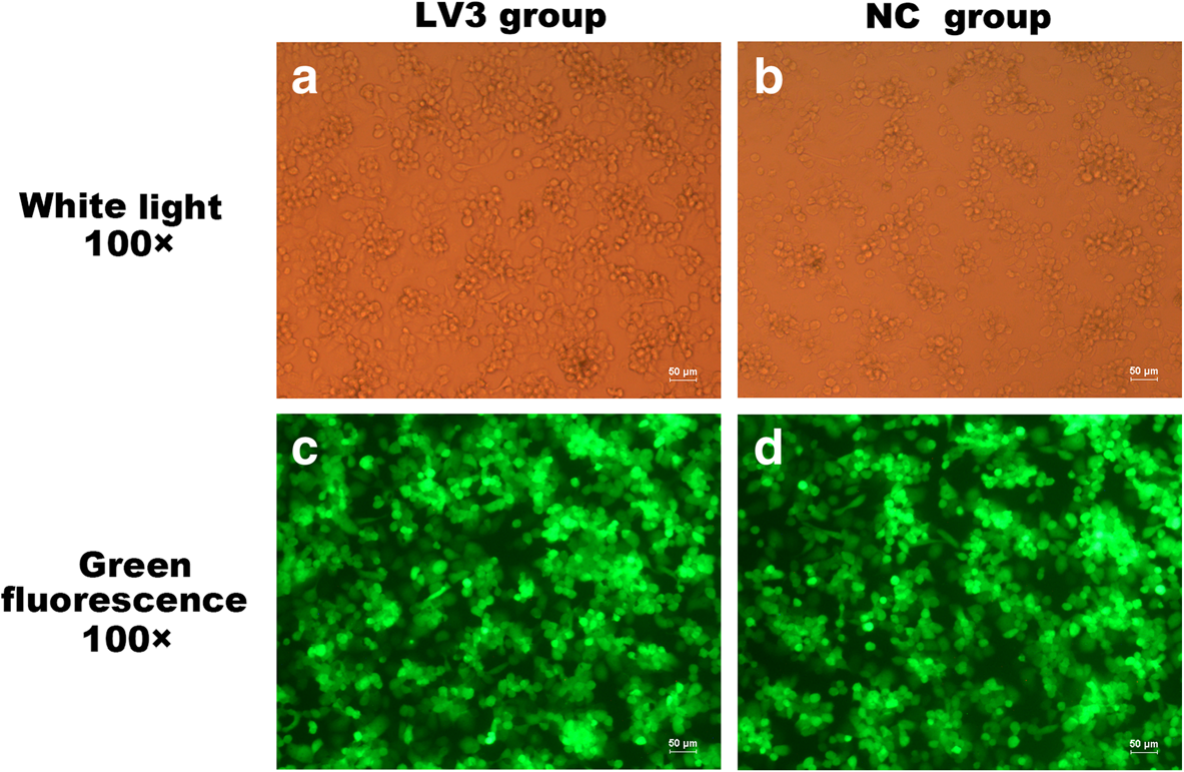
Knocking down ARRB2 expression by siRNA. siRNA targeting ARRB2 (LV3-β-arrestin2, 5′-GGACACCAACCTCATTGAATT-3′) and its empty vector (LV3-NC, 5′-TTCTCCGAACGTGTCACGT-3′) were transfected into THP-1 monocytes. After that, cells were subsequently differentiated into macrophages with 1.28 μM of PMA (5 × 10^5^ cells/well) for 48 h. The transfection efficiency of each group was detected by a fluorescence microscope (×100). **a**–**b** The white-light photographs of monocyte-derived macrophages which are transfected with the ARRB2 downregulation siRNA (LV3-ARRB2) and the negative control siRNA (LV3-NC). **c**–**d** The fluorescence photographs of monocyte-derived macrophages which are stably transfected with LV3-ARRB2 and LV3-NC

**Fig. 6:**
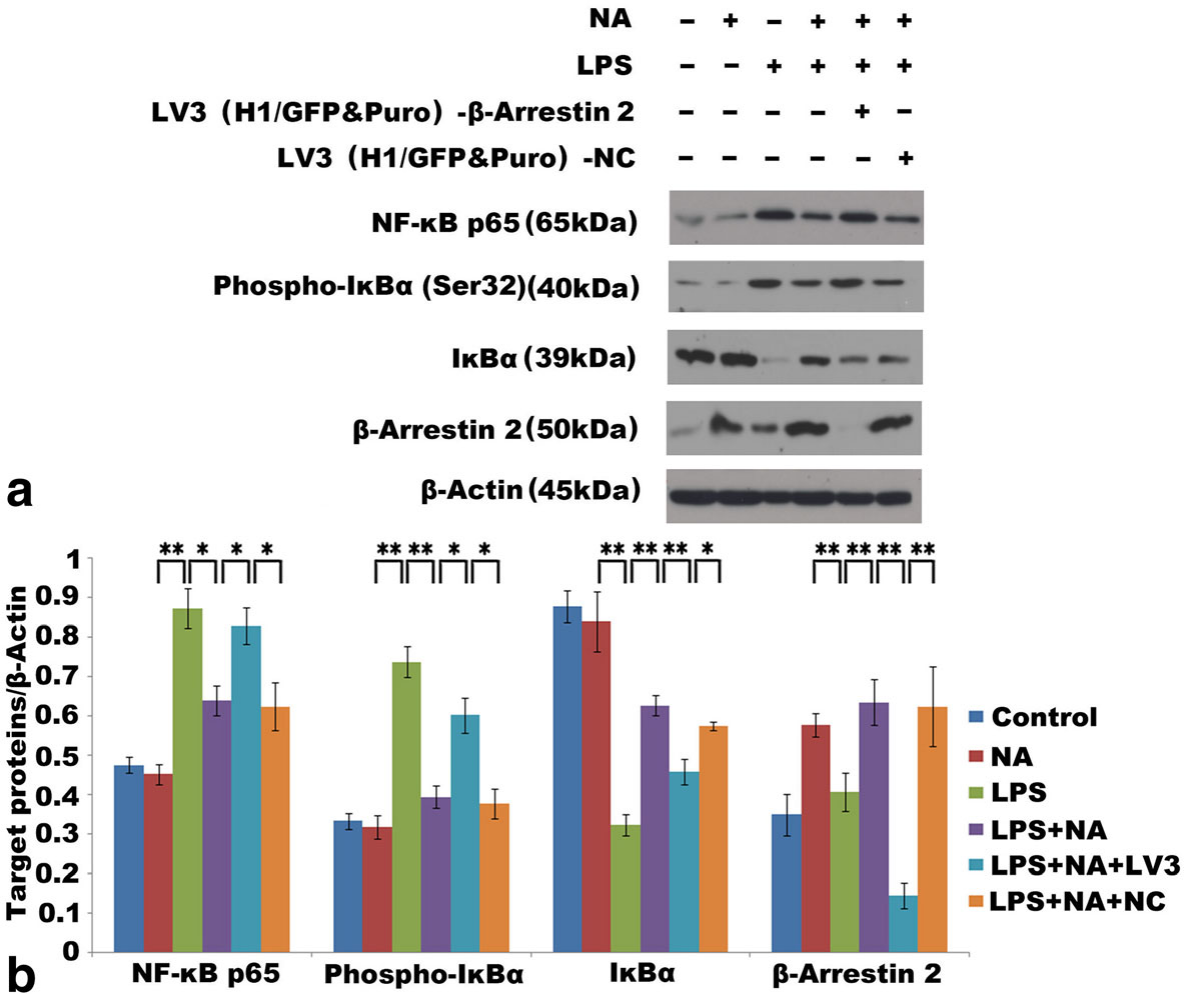
Deficiency of ARRB2 restrained adrenergic-mediated inhibition of the phosphorylation activity of NF-κB signaling pathway. NA (100 μM) stimulation was applied to simulate the state of sympathetic pathway activation. **a** Representative western blot image showing the expression of NF-κB p65, IκBα, phosphor-IκBα, and ARRB2 in each group. **b** Densitometry analysis shown as graph bars for each protein expression normalized with β-actin. Increased expression of NF-κB p65 and phosphor-IκBα, and decreased expression of IκBα were detected in the LPS-stimulation group. Adrenergic-stimulation significantly inhibited LPS-induced NF-κB p65 and phosphor-IκBα expression. Meanwhile, increased expression of ARRB2 and IκBα was detected. Deficiency of ARRB2 significantly increased phosphor-IκBα and NF-κB p65 expression, and decreased IκBα expression in spite of adrenergic-stimulation. Each experiment was independently replicated three times. *P < 0.05 and **P < 0.01 by one-way ANOVA

**Fig. 7:**
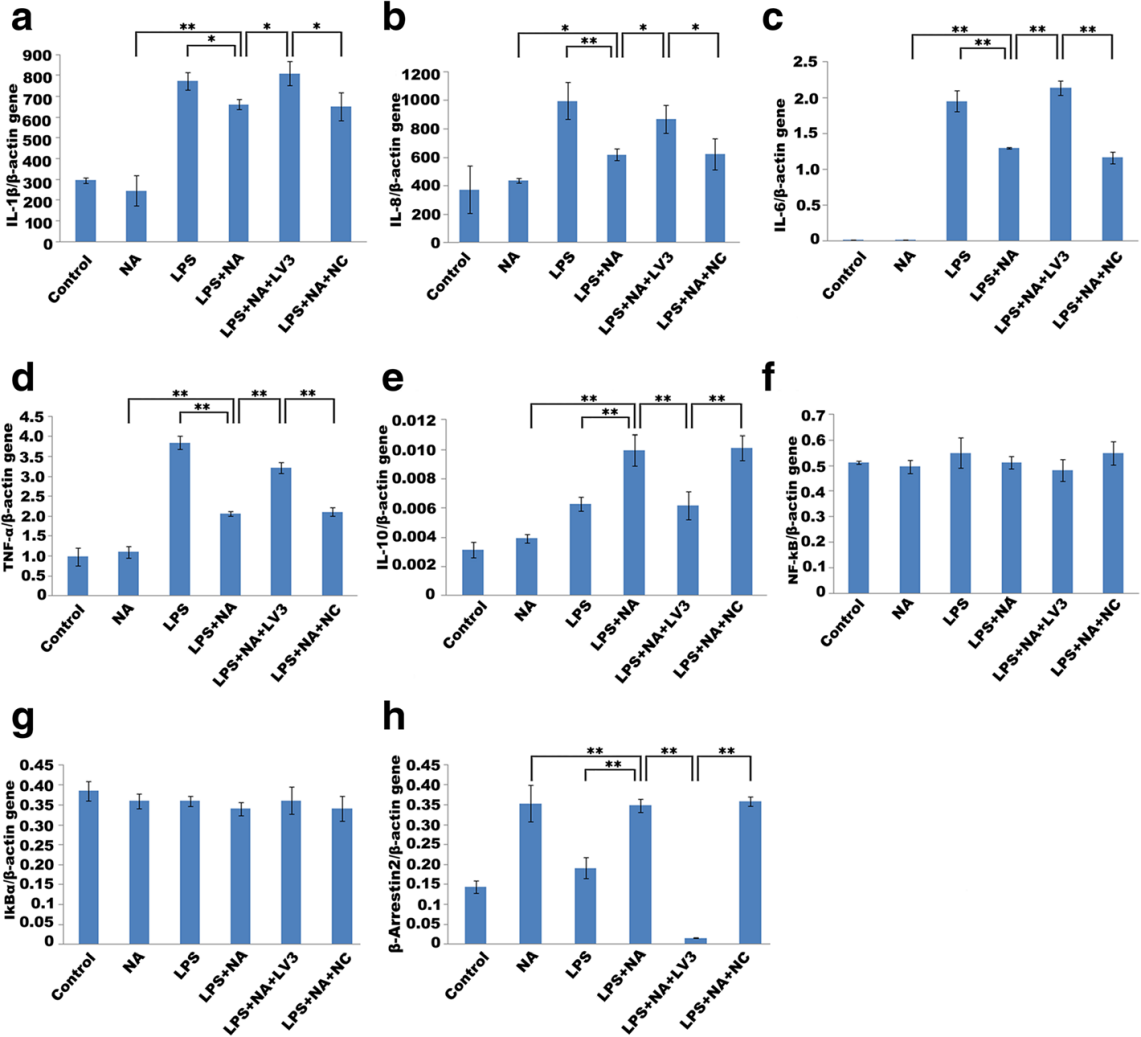
Effect of deficiency of ARRB2 on adrenergic-mediated alteration of NF-κB signaling activity and cytokine expression detected at gene level. LPS induced high gene expression of IL-1β (**a**), IL-8 (**b**), IL-6 (**c**), and TNF-α (**d**). Adrenergic-stimulation considerably declined LPS-induced cytokine gene expression (**a**–**d**). Moreover, IL-10 gene expression was significantly increased by adrenergic-stimulation (**e**). However, deficiency of ARRB2 significantly increased the gene expression of IL-1β (**a**), IL-8 (**b**), IL-6 (**c**), and TNF-α (**d**), and decreased IL-10 gene expression in spite of adrenergic stimulation. On the other hand, the gene expression of ARRB2 was significantly descended with the transfection of LV3-ARRB2 (**h**). The gene expression of NF-κB and IκBα was not affected by siRNA transfection (**f**–**g**). Data represents each mRNA level relative to β-actin. Each experiment was independently replicated three times. *P < 0.05 and **P < 0.01 by one-way ANOVA

To investigate the exact function of ARRB2 in the β-adrenoreceptor-mediated immunodepression, the expression of ARRB2 was developed by its siRNA in THP-1 monocytes. A strong green fluorescent signal after transfection was detected by a fluorescence microscope to confirm high transfection efficiency of siRNA (see Fig. 5). Moreover, the results of both western blot and PCR analysis revealed that the expression of ARRB2 was remarkably reduced by the treatment of ARRB2 siRNA (*P* < 0.01, *P* < 0.01, see Figs. 6a–b and 7h).

### Deficiency of ARRB2 attenuates the adrenergic mediated inhibition of NF-κB signaling pathway

Adrenergic stimulation remarkably suppressed LPS-induced activation of NF-κB signaling pathway by decreasing the expressions of NF-κB p65 and phosphor-IκBα, while IκBα expression was increased. Meanwhile, ARRB2 expression was drastically increased (see Fig. 6a,b). With deficiency of ARRB2, the expressions of NF-κB p65 and phosphor-IκBα were markedly increased (*P* < 0.05, *P* < 0.05), while the IκBα expression was significantly decreased (*P* < 0.01) in spite of adrenergic stimulation. Moreover, the control of siRNA had no influence on the expression of each factor as displayed in Fig. 6a,b. On the other hand, there was no significant difference in gene levels of NF-κB and IκBα among the available groups in PCR analysis (see Fig. 7f,g).

### Deficiency of ARRB2 reverses adrenergic-mediated monocyte dysfunction

**Fig. 8:**
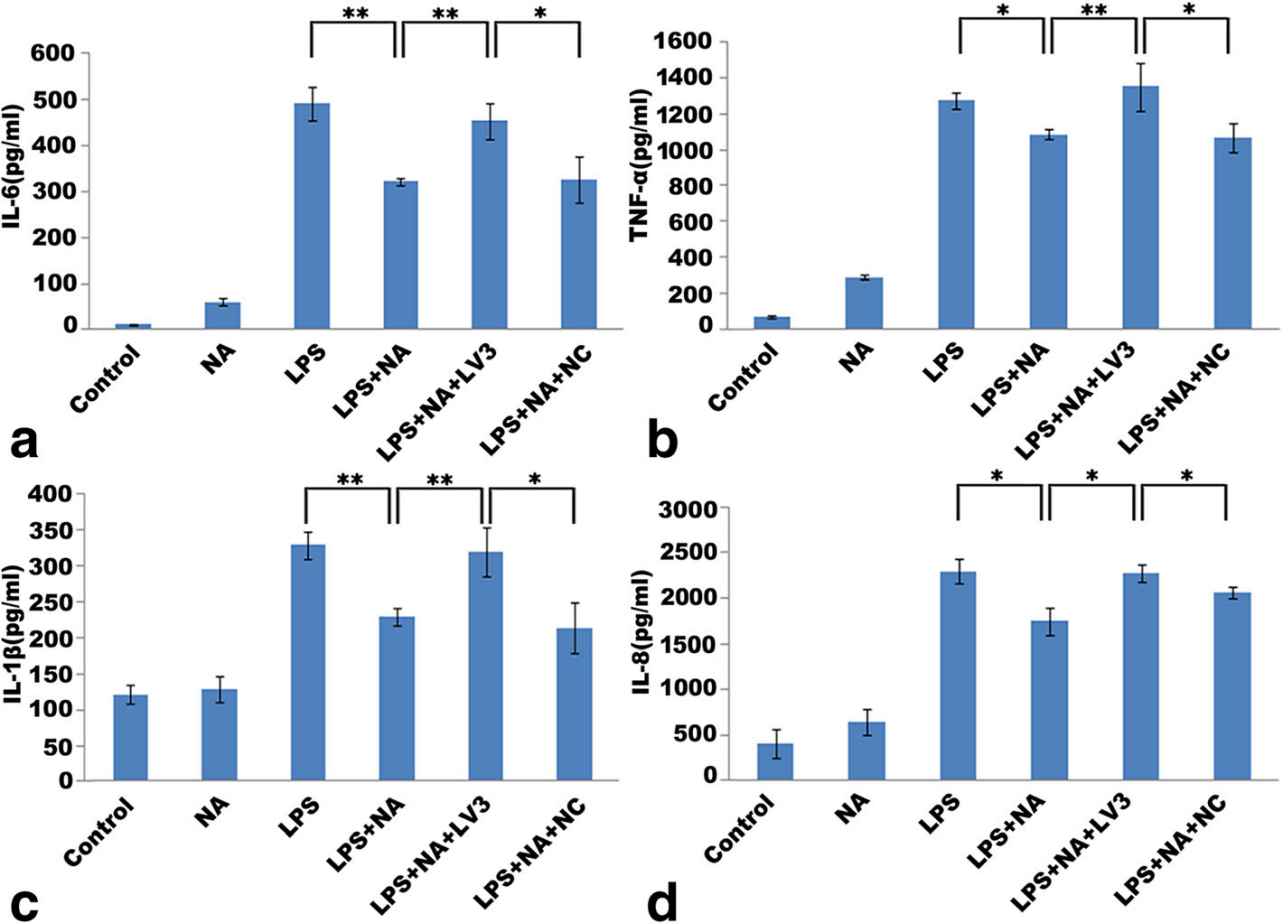
Deficiency of ARRB2 significantly reverses adrenergic-stimulated monocyte dysfunction. The levels of cytokine release were detected by ELISA. LPS induced high release of IL-6 (**a**), TNF-α (**b**), IL-1β (**c**), and IL-8 (**d**). Adrenergic-stimulation significantly inhibited LPS-induced pro-inflammatory cytokine release including IL-6 (**a**), TNF-α (**b**), IL-1β (**c**), and IL-8 (**d**). However, deficiency of ARRB2 substantially reversed the adrenergic suppression in cytokine release by elevating IL-6 (**a**), TNF-α (**b**), IL-1β (**c**), and IL-8 (**d**) levels. Each experiment was independently replicated three times. *P < 0.05 and **P < 0.01 by one-way ANOVA

As displayed in Fig. 8, LPS-induced activation of monocytes resulted in the high expression of pro-inflammatory cytokines including IL-6, TNF-α, IL-1β, and IL-8. Adrenergic-stimulation notably decreased LPS-induced secretion of these cytokines (*P* < 0.01, *P* < 0.05, *P* < 0.01, and *P* < 0.05). With deficiency of ARRB2, these cytokine levels were substantially elevated in spite of adrenergic stimulation (*P* < 0.01, *P* < 0.01, *P* < 0.01, and *P* < 0.05). In addition, the control of siRNA had no impact on the expression of each cytokine (see Fig. 8a–d). Consistently, deficiency of ARRB2 considerably increased the gene expression of these cytokines in spite of adrenergic stimulation (*P* < 0.01, *P* < 0.01, *P* < 0.05, and *P* < 0.05) (see Fig. 7a–d). Moreover, adrenergic-induced IL-10 gene level elevation was remarkably inhibited by deficiency of ARRB2 (*P* < 0.01, see Fig. 7e).

## Discussion

SIDS remains under intensive investigation as it substantially contributes to potential repair mechanisms of the brain and increased risk of infections after stroke [6, 25]. In this study, the in vivo results demonstrate a profound stroke-induced splenic monocyte dysfunction characterized by reduced pro-inflammatory cytokine release and increased anti-inflammatory cytokine production. Besides, the achieved results reveal that stroke induces increased splenic ARRB2 expression that has a significant positive correlation with the sympathetic system activity. Moreover, blockade of the sympathetic system by propranolol prominently reverses a stroke-induced immunodepression symptom as well as splenic ARRB2 expression. Additionally, a recent study has demonstrated that splenic immunity in the group of propranolol + sham is not different from the group of vehicle + sham, reflecting that monotherapy of propranolol may have no influence on splenic immunity [26]. In summary, these findings suggest that stroke-induced activation of the sympathetic system significantly contributes to splenic ARRB2 expression and monocyte dysfunction. However, it still remains indistinct whether ARRB2 functions as a regulator in sympathetic-triggered splenic monocyte dysfunction after stroke.

Regarding the in vitro study, ARRB2 expression was knocked down in monocytes in order to investigate its function on sympathetic-triggered SIDS. The obtained results reveal that adrenergic-stimulation significantly promotes ARRB2 expression and induces profound monocyte dysfunction. Nevertheless, deficiency of ARRB2 prominently reverses adrenergic-mediated inactivation of monocytes. Hence, ARRB2 is recommended to be involved in adrenergic-mediated monocyte dysfunction. In addition, it was attempted to further explore the effect of ARRB2 on the activity of NF-κB signaling as NF-κB plays a key role in regulating immune responses [27]. Moreover, deficiency of ARRB2 significantly reverses adrenergic-inhibition on the activity of NF-κB signaling. Therefore, ARRB2 is regarded as a key intracellular mediator transmitting the adrenergic activity to intracellular factors such as NF-κB.

Currently, ARRB2 is generally reported to be implicated in multifarious physiological and pathophysiological processes. A previous study found that ARRB2 was elevated in cardiomyocytes in response to cardiac ischemia-reperfusion (I/R) injury and accounted for I/R-induced cardiomyocyte death [28]. Another study declared that increased ARRB2 in infiltrated macrophages after myocardial infarction (MI) plays a protective role in MI-induced inflammation [29]. Similarly, ARRB2 was found to negatively regulate inflammation response in the setting of polymicrobial infections and sepsis [30, 31]. Hoffmann et al. [32] further emphasized the significant role of ARRB2 on GPCRs signaling by indicating a rapid two-step binding as well as activation process between GPCRs and ARRB2. Even though the multiple functions of ARRB2 have been explored in cardiac diseases and infections, very limited information is available about alterations in the expression and functions of ARRB2 after stroke. On the other hand, the endogenous factors involved in the sympathetic pathway that mediates SIDS remain poorly understood. In the present study, for the first time, we demonstrated that elevated ARRB2 expression in response to stroke-induced sympathetic hyperactivity was deeply involved in the sympathetic-triggered monocyte dysfunction. These novel findings further intensify the concept of the negative regulatory function of ARRB2 in the inflammation response. Moreover, the results contribute towards a further understanding of the mechanism underlying the sympathetic pathway that mediates SIDS, paving a way to provide a new clue and experimental sustainment for achieving therapeutic target.

Additionally, previous experimental and clinical studies demonstrated that the sympathetic system was a major mediator of the brain-immune interaction and suggest that brain lesions, especially severe brain injury, cause an increase in the release of catecholamines, which restrain various peripheral immune cell functions [11, 33, 34]. The recruitment of these impaired cells to the injured tissue probably protects the brain tissue from secondary inflammatory injury [35, 36]. Unexpectedly, it was disclosed that the infarct volume and neurological deficit were significantly improved by propranolol administration. Romer et al. [13] reported the reduction of the infarct volume after blocking the SIDS by inhibiting the sympathetic nervous system. However, the achieved results further clarify that neither the infarct volume nor neurological deficit have a positive correlation with ARRB2 expression. Accordingly, adrenergic-induced ARRB2 is recommended to be involved in SIDS rather than reduced infarct volume.

Strategies targeting post-stroke inflammatory reactions were studied in several previous researches, in order to improve the prognosis of stroke patients [3, 37]. Exploration of the endogenous regulator and immunosuppressive signaling factors may contribute to exploiting new therapeutic targets for stroke and its complications. The present study investigated a novel function of ARRB2 that was playing a dominant role in stroke-induced immunodepression. Hence, ARRB2 might be a promising therapeutic target for the management of stroke. The limitation of this study is mainly attributed to in vitro experiments and individual immune cells. Thus, subsequent in vivo and in vitro studies based on other cell populations, such as T cells and microglial cells, will be required to further confirm the role of ARRB2 in post-stroke inflammatory reactions. In addition, it is recommended that propranolol plus sham-operation group be designed in future in vivo studies to explore the potential effect of propranolol monotherapy on the variables.

## Conclusions

In this study, the obtained results indicate that splenic ARRB2 is elevated after stroke due to hyperactivation of the sympathetic system. Moreover, ARRB2 functions as a key regulator of the adrenergic-mediated inflammatory response. Accordingly, ARRB2 may be a promising therapeutic target for the immunological management of stroke in clinic.

## Abbreviations

ARRB2: Beta-arrestin2
BCA: Bicinchoninic acid
GPCRs: G-protein-coupled receptors
I/R: Ischemia-reperfusion
IACUC: International Animal Care and Use Committee
IL-10: Interleukin-10
IL-1β: Interleukin-1β
IL-6: Interleukin-6
LPS: Lipopolysaccharide
MCAO: Middle cerebral artery occlusion
MI: Myocardial infarction
PMA: Phorbol myristate acetate
PMSF: Phenylmethane sulfonyl fluoride
PVDF: Polyvinylidene difluoride
RIPA: Radio-immunoprecipitation assay
SIDS: Stroke-induced immunodeficiency syndrome
TBST: Tris-buffered saline Tween-20
TNF-α: Tumor necrosis factor-α
TTC: 2,3,5-triphenyltetrazolium chloride
